# Hidden Drug Resistant HIV to Emerge in the Era of Universal Treatment Access in Southeast Asia

**DOI:** 10.1371/journal.pone.0010981

**Published:** 2010-06-08

**Authors:** Alexander Hoare, Stephen J. Kerr, Kiat Ruxrungtham, Jintanat Ananworanich, Matthew G. Law, David A. Cooper, Praphan Phanuphak, David P. Wilson

**Affiliations:** 1 National Centre in HIV Epidemiology and Clinical Research, The University of New South Wales, Sydney, Australia; 2 The HIV Netherlands Australia Thailand Research Collaboration, The Thai Red Cross AIDS Research Centre, Bangkok, Thailand; 3 Faculty of Medicine, Chulalongkorn University, Bangkok, Thailand; McGill University AIDS Centre, Canada

## Abstract

**Background:**

Universal access to first-line antiretroviral therapy (ART) for HIV infection is becoming more of a reality in most low and middle income countries in Asia. However, second-line therapies are relatively scarce.

**Methods and Findings:**

We developed a mathematical model of an HIV epidemic in a Southeast Asian setting and used it to forecast the impact of treatment plans, without second-line options, on the potential degree of acquisition and transmission of drug resistant HIV strains. We show that after 10 years of universal treatment access, up to 20% of treatment-naïve individuals with HIV may have drug-resistant strains but it depends on the relative fitness of viral strains.

**Conclusions:**

If viral load testing of people on ART is carried out on a yearly basis and virological failure leads to effective second-line therapy, then transmitted drug resistance could be reduced by 80%. Greater efforts are required for minimizing first-line failure, to detect virological failure earlier, and to procure access to second-line therapies.

## Introduction

HIV/AIDS arose in Asia in the early-to-mid 1980s. By the 1990s HIV epidemics had established in numerous countries; among the worst affected were Thailand and Cambodia with HIV prevalence levels of 1–2%. Currently Thailand, Cambodia, and Myanmar have been experiencing declines in HIV prevalence [1], [2], however, countries such as Vietnam, Indonesia, Pakistan and China have observed growth in their epidemics [3].

Effective antiretroviral therapy (ART) is currently being scaled up in most countries in the region. In principle, anyone who is treatment eligible, according to country-specific guidelines but generally similar to the WHO treatment guidelines for resource limited settings [4], can receive ART to slow disease progression [5]. But with greater treatment coverage there is concern about the development of drug resistance, especially in countries where second-line therapy is not widely available. The transmission of drug-resistant strains can potentially lead to ineffective treatment for individuals [6] and reduce their treatment options.

Transmitted drug resistance is a problem around the world, including the Southeast Asia region. Documented rates of transmitted drug resistance include 4% in 2003–2004 in Japan [7] and increases in Taiwan from 6.6% in 1999–2003 to 12.7% in 2003–2006 [8] and Thailand from <1% in 2003 to 5.2% in 2006 [9]. The vast majority of patients (∼80%) in Asia start treatment on AZT/d4T plus 3TC plus EFZ/NVP [10]. This regimen is likely to be the standard for the foreseeable future (perhaps with tenofovir replacing AZT/d4T). If mutations that confer resistance to this standard regimen become widespread, ART rollout strategies could be compromised in a way that is not seen in developed countries with more treatment options.

The primary means to detect transmitted drug resistance is to perform blood tests on newly infected treatment-naïve individuals. Resistance strains can be divided up into two broad categories, namely, majority-resistant and minority-resistant variants. Majority resistant strains are detected through conventional nucleotide sequencing methods after polymerase chain reaction (PCR) amplification, however, these methods are not sensitive enough to detect minority-resistant strains that comprise less than ∼25% of the viral population [11]. These minority-resistant variants can be detected using advanced real time PCR assays [12], [13]. There is potential for these minority strains to go undetected in the population, leading to under-estimates of transmitted resistance levels.

We sought to estimate the potential levels of acquired and transmitted (majority and minority) drug resistant strains of HIV that could emerge in a typical Southeast Asian population. We do this through the development of a biologically realistic mathematical transmission model. We use the situation in Thailand as a representation for a general Asian epidemic and thus calibrated the model to reflect the epidemic in Thailand. Thailand is a leading example of treatment scale-up with the introduction of ART through the National Access to Antiretroviral Program for People who have AIDS by the Ministry of Public Health Access to Care program [14], [15] and extended to the government's National AIDS Program by the National Health Security Office in 2004 [16]. Our mathematical model is parameterized using specific clinical, demographic, biological, and behavioral data in and around Bangkok, Thailand, before second-line therapy became available. Although second-line therapy is rolling out in Thailand, it is not available for many HIV-infected people in other countries. Our model extends previous mathematical models of HIV drug resistance applied to other settings (e.g. [17], [18], [19], [20]) and models that incorporate at-risk groups for the Southeast Asian setting [21].

## Methods

**Figure 1:**
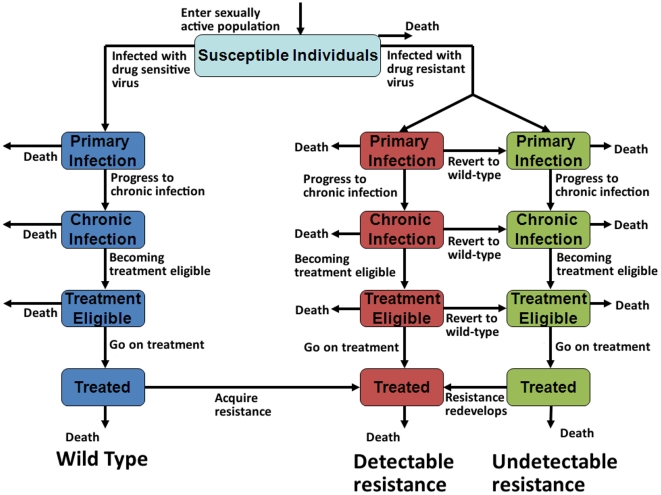
Schematic diagram of our mathematical model. The natural progression of HIV infection captured by our model, with disease progression illustrated vertically; the model is also divided into three arms: each arm governs a different type of virus (wild-type, majority-resistant variants, minority-resistant variants).

Our model describes the unique nature of Asian HIV epidemics whereby epidemics typically emerge and are initially driven by injecting drug use and sex work. Waves of infection occurred in these population groups, followed by infection among clients of sex workers and their regular sexual partners which led to generalized epidemics. In recent years HIV epidemics have emerged among men who have sex with men. This epidemic pattern has been observed in numerous Southeast Asian countries [22], [23] and is captured by our model (see Figure S1). To reflect disease progression, we assumed that all HIV-infected people progress from primary/acute HIV infection, to chronic/asymptomatic infection, to a treatment-eligible stage, and then may receive treatment (Fig. 1). Each disease stage is associated with a different viral load and hence a different level of infectiousness [24], [25]. Disease progression rates are assumed to be different in the presence of a majority-resistant strain due to lower viral fitness, but we assume minority-resistant strains have the same fitness as wild-type virus. We assume that reduced viral fitness of majority-resistant strains diminishes their replicative capacity and thus their ability to be transmitted. A multiplying factor was used to model a decrease in viral fitness between 5% and 50% [18], [19]. Mathematical Details S1 contains more details about the implementation of this viral fitness factor. Once on treatment, we assume that patients will continue using their ART regimen, even if treatment failure occurs, as limited second or third line treatment options are available in many settings.

The level of adherence to ART is associated with clinical success [26], [27] as systemic drug concentrations determine the degree of pressure to select for drug resistant strains [28], [29], [30], [31], [32]. Although there is variability in adherence between people, in our model we do not explicitly model adherence to ART but based on international clinical data [33], [34], [35] we assume that 3–5% of people on first-line ART select for drug resistant mutations each year and acquire drug-resistant strains. We track populations of people infected with either wild-type HIV or strains of drug-resistant HIV that are detectable or appear to have reverted to wild-type. Those people who have strains that appear to revert to wild-type have minority-resistant variants and it is assumed that majority-resistant variants will quickly emerge under pressure of ART. We use our model (and uncertainty and sensitivity analyses [36], [37]) to estimate the future trajectories of wild-type and drug-resistant HIV epidemics, determine the biological, clinical, and behavioral factors that are most important in giving rise to these evolving epidemics and how they might change with time in order to plan public health prevention and clinical practice strategies most appropriately. Some mathematical modeling has been carried out to forecast HIV epidemics in Southeast Asia [21], but no previous model has investigated the impact of drug resistance in this region.

The model was then used to assess the impact of regular viral load testing in a setting where second line treatment is available and commenced once virological failure is detected. We assumed that viral load tests could be performed at regular intervals on all those who are receiving treatment. We simulated different scenarios of frequency of viral load testing: once every 2 years, every year, twice yearly, or quarterly. We also assumed that a period of one week was required between the time of the test and receiving the test results and starting the patient on effective second-line treatment. Full technical detail of the model structure, assumptions and parameter values can be found in the supporting information.

## Results

### Emergence of Drug Resistance

**Figure 2:**
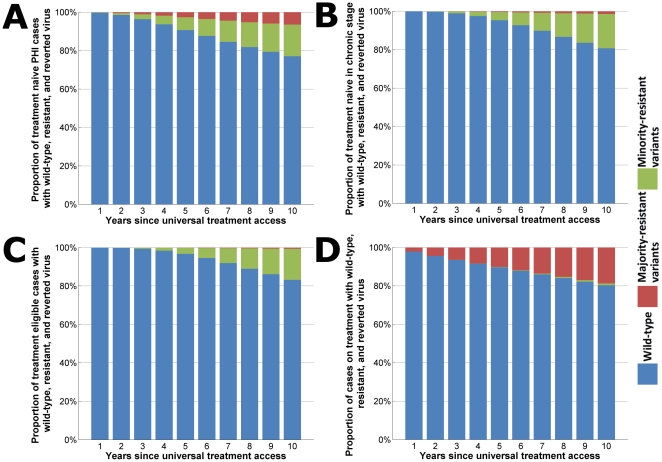
Stacked column charts indicating proportions of HIV viral types. Proportions of all HIV infections that are predominantly wild-type virus (blue), drug-resistant strains that are undetectable/minority-resistant variants (green), or drug-resistant strains that can be detected/majority-resistant variants (red) for HIV-infected cases in (**a**) primary infection, (**b**) chronic infection, (**c**) treatment-eligible stage, and (**d**) on treatment. Plots are over the time period since the introduction of universal access, and without any options for second-line therapy.

After 10 years of universal ART without access to any second line therapies, moderately high levels of drug resistance can be expected in the HIV-infected population. People on ART will start to acquire drug resistant strains of virus. If second and subsequent lines of therapy are not widely available and failed regimens continue to be used then the emergent drug-resistant strains can be transmitted to susceptible individuals. Subsequently, the proportion of newly-infected treatment-naïve HIV cases that have drug-resistant strains could be substantial. Our model estimates that after 10 years of universal ART without monitoring of treatment failure and optimizing therapy ∼24% of new infections could include drug-resistant mutations (Fig. 2a). Approximately one-third of cases in the primary/acute stage of infection with drug-resistant mutations could have majority-resistant variants of HIV that are detectable and the remainder would have minority-resistant variants (Fig. 2a).

### Most subjects infected with transmitted resistant virus appear to revert to wild type

In the absence of the pressure of ART, majority-resistant strains of HIV tend to revert to become minority-resistant variants that appear to be exclusively wild-type and not detected by standard sequencing methods. According to our model, after 10 years of universal access to ART without second-line options ∼20% of treatment-naïve cases in asymptomatic stage would have some drug-resistant strains and ∼17% of cases at treatment-eligible stage of infection would have some drug-resistant strains (Fig. 2b, c). However, it is likely that the vast majority of these cases would have minority-resistant variants: only ∼1% and <1% of the respective HIV cases would have detectable majority-resistant variants after 10 years (Fig. 2b,c). Thus, drug-resistant HIV could remain hidden and will only re-emerge when selective pressure of ART is applied. Of course, the rate of reversion could differ between different antiretroviral drug-based mutations. The re-emergence of drug-resistant strains could be quick once treatment is commenced by individuals. The vast majority (∼95%) of individuals on ART who have drug-resistant strains would have majority-resistant variants (Fig. 2d). Based on our model we estimate that after 10 years of universal treatment access ∼20% of all people that are on ART would have drug-resistant strains of HIV (Fig. 2d).

### Factors determining the prevalence of drug resistance

**Figure 3:**
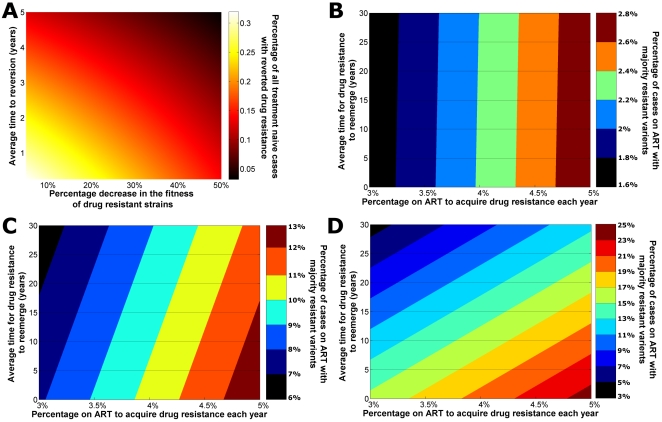
Series of response surfaces from sensitivity analyses. (**a**) A response surface plot of the proportion of treatment-naïve HIV-infected cases with minority-resistant variants versus viral fitness of drug-resistant strains and the average time for majority-resistant variants to revert to minority-resistant variants in the absence of ART. (**b**)–(**d**) Contour plots of the proportion of cases on ART that have majority-resistant variants (colored contours) versus the rate at which people infected with wild-type acquire drug resistant virus (x-axis) and the average time for majority-resistant variants to emerge for people infected with minority-resistant variants (y-axis) after (**b**) 1 year, (**c**) 5 years, and (**d**) 10 years of universal treatment access.

Key factors giving rise to the prevalence of drug resistance differ between populations of treatment-naïve and treatment-experienced individuals. Multivariate sensitivity analyses revealed that the average time for resistant strains to appear to revert to wild-type virus and the relative fitness of drug-resistant strains were the most important parameters for determining the prevalence of majority-resistant variants in treatment-naïve cases (Fig. 3a). The relative fitness of viral strains with resistant mutations is a key determinant in the prevalence of transmitted drug resistance. The greater the fitness of these strains the larger the prevalence of ‘hidden’ resistance in the treatment-naïve population. Transmitted drug resistance increases with fitter drug-resistant strains and slower majority-to-minority variant reversion times. In contrast, the average time for drug-resistant strains to re-emerge upon pressure of ART (in individuals with minority-resistant variants; that is, to become majority-resistant variants upon applying pressure of ART) and the percentage of patients that acquire drug resistance per year (in individuals with wild-type) were found to be the most important factors in determining the proportion of treated individuals with majority-resistant variants (Fig. 3b–d). Interestingly, the relative importance of these two factors changes over time. To illustrate this, in Figure 3b–d we present a series of contour plots of the prevalence of majority-resistant variants among the treated population after 1 year (Fig. 3b), 5 years (Fig. 3c), and 10 years (Fig. 3d) after commencing universal treatment access. We found that the number of people receiving treatment that have detectable drug resistance after one year of universal access to treatment is almost completely dependent on the percentage that acquire resistance per year, as indicated by the close to vertical lines in Figure 3b. After five years, the dependence has begun to shift such that the average time for resistance to reemerge begins to have an impact on the prevalence of drug-resistant HIV (Fig. 3c). After 10 years, the prevalence of detectable drug resistance is now more dependent on the average time for drug resistance to reemerge for transmitted drug-resistant strains than on the rate of acquired resistance (Fig. 3d). When projected even further, after 20 years the vast majority of drug-resistant cases are due to transmitted resistance (see Figure S2). This suggests that the nature of the drug-resistant HIV epidemic could change considerably, initially being driven by acquired resistance and then evolve to be dominated by cases who have transmitted (but hidden) drug resistance.

### Reducing transmitted drug resistance through viral load testing

**Figure 4:**
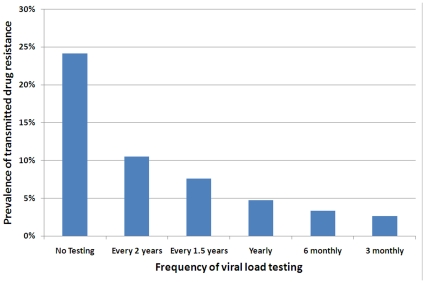
Prevalence of transmitted drug resistance after 10 years with various viral load testing frequencies. Testing scenarios include: no testing, once every two years, once every 1.5 years, yearly, twice yearly, and quarterly. Once tested, it is assumed that anyone failing treatment is taken off the failed regimen and given access to new treatment.

In many Southeast Asian countries, treatment failure is often realized due to clinical symptoms rather than the presence of mutations or virological or immunological failure. Frequent viral load testing is generally infeasible due to financial constraints. However, viral load testing for monitoring patients' responses to ART is available in some settings and it could be expected that it will become more common across the region in the future. Therefore, we used our model to estimate the expected proportion of newly acquired HIV infections to have drug-resistant strains versus the frequency of viral load testing of individuals on ART (assuming that treated cases that experience virological failure commence and are maintained on second and subsequent lines of therapy that successfully suppresses viral load). In Figure 4 we present the expected levels of transmitted drug resistance versus the frequency of viral load testing. As the testing frequency is increased, a substantial reduction in the prevalence of transmitted drug resistance is observed. Providing a test every two years will reduce the prevalence by more than 50% compared to no viral load testing. With yearly testing, the proportion of all new infections with transmitted resistance drops below 5% (that is, an 80% relative reduction). According to our model, if viral load testing is further increased to every three months, transmitted drug resistance will make up only ∼2.5% of all infections (reducing transmitted resistance by 90% compared to the situation where no testing is carried out).When compared to yearly testing, our model found that six and three monthly testing offered a relative reduction of 28% and 44% in transmitted drug resistance levels, respectively.

## Discussion

Effective treatment with antiretroviral drugs reduces viral load which improves the health of treated individuals and also decreases infectiousness and the potential to transmit the virus to others [24], [25], [38]. However, persons infected with drug-resistant HIV have reduced therapeutic options for their survival [39], [40]. Antiretroviral resistance was detected against the first drug used against HIV, AZT, shortly after it was introduced [41]. Subsequently, resistance to every currently licensed antiretroviral drug has been observed. Drug-resistant strains of HIV that are acquired through use of ART can then be transmitted to susceptible people. The first report of observed transmission of drug-resistant HIV was in 1993 [42]. The transmission of drug resistance is becoming an increasing problem among many nations with long histories of ART. Data on rates of transmitted and acquired resistance in Southeast Asian countries is limited. In the few areas in which HIV transmitted resistance have been measured in Asia, already moderate levels (∼4–5%) have been observed in some countries [43], [44], [45]. In other regions of the world, prevalence of drug resistant HIV among treatment-naïve persons has been estimated to be up to 25% [46]. It is important to implement strategies in Southeast Asian countries to avoid the high prevalence of transmitted drug resistance that has occurred elsewhere.

We demonstrated that if treatment options are limited for those who fail first-line therapy then the prevalence of acquired and transmitted drug resistant strains of HIV could be relatively large. The prevalence of transmitted drug resistance could be ∼24% after ten years of universal treatment access if there is no viral load monitoring and access to second-line therapy. However, most (99%) of the drug resistance could remain ‘hidden’ as minority-resistant variants that are not detectable by standard sequencing methods. Majority-resistant variants are likely to emerge at significantly faster rates than expected once treatment is initiated [47]. While there is some uncertainty about whether minority-resistant strains have a substantial [12], [48] or limited [13] impact on the success of antiretroviral therapy, the impact of majority-resistant strains on treatment is known to be significant. Majority-resistant strains may be more likely to survive in the presence of antiretroviral therapy than wild-type strains, however, they are likely to have reduced replicative capacity leading to lower viral loads in plasma and genital fluids and thus lower potential to be transmitted to other people. Our model demonstrated the importance of viral fitness whereby strains with higher fitness are more likely to lead to higher population levels of transmitted drug resistance (Figure 3a).

To reduce the prevalence of drug resistance among treatment-naïve individuals it is recommended that treated cases are regularly monitored and that second-line and subsequent lines of therapy are made available for those who have failed first-line regimens. We investigated the expected impact on transmitted drug resistance of different frequencies of viral load monitoring and access to second-line therapy when required. Even with a modest testing frequency of once every two years for patients on ART, the model demonstrates a large reduction in the amount of transmitted drug resistance would be achieved. Testing as frequently as quarterly could reduce the prevalence of transmitted drug resistance by ∼90%. In Thailand, since 2008 second-line therapy with TDF/3TC/LPV/r has been widely available as well as once yearly viral load monitoring and genotyping (for those with viral load of more than 2000 copies per ml). However, there are limited treatment options in Thailand and patients with TDF resistance will have difficulties in finding effective second line treatment options. Wide availability of third line treatments for patients in this region will be unlikely in the near future. Therefore, it is highly important to minimise drug resistance. Based on our model, yearly testing can reduce transmitted drug resistance to below 5%. It is important for countries in Southeast Asia to procure access to second-line therapies and determine ways of implementing regular viral load monitoring. It will then be important to procure third-line and salvage therapies for patients in this region, however, this is unlikely to be feasible in the near future. Viral load testing is not widely available in many Asian countries and the emergence of drug resistant HIV is not typically assessed during patient consultation [49], [50]. Without viral load or genotypic monitoring, late detection of treatment failure may facilitate the acquisition of numerous additional resistance mutations [51]. Monitoring of patients' CD4 counts and viral load levels is being carried out in the Treat Asia HIV Observational Database (TAHOD) study [52]. TAHOD and other surveillance activities such as the Treat Asia Studies to Evaluate Resistance (TASER) study are important foundations for monitoring treatment success and detecting the development of resistance to antiretrovirals. In some countries governments pay for the first triple combination, but patients pay for other drugs if the first regimen fails. This barrier to accessing second-line therapy needs to be overcome else persistent use of sub-optimal or failed regimens will occur. Continued use of a failed regimen may select for increases in drug-resistant HIV strains that may then be transmitted to others.

Limited combinations of antiretrovirals are available for first-line treatment in most Southeast Asian countries. In Thailand, first-line therapy is based on NNRTIs and usually consists of a fixed dose combination of d4T/3TC/NVP, with a newer regimen of ZDV/3TC/NVP recently rolled out [53]. The prevalence of resistance in Thailand to NNRTI and NRTI based drug combinations can restrict second-line options in close to half of patients [54]. The World Health Organization has recently made recommendations against use of Triomune (d4T/3TC/NVP) in initiation of first line therapy [55], [56]. New treatment guidelines for Thailand will also be released shortly [57]. These guidelines recommend AZT- and TDF- with EFV or NVP and 3TC as preferred first-line. There is a planned 2-year phase out of d4T for patients already receiving d4T. Similar clinical approaches may not be achievable in all resource-limited settings and the use of Triomune is likely to continue. Obtaining access to more first-line antiretroviral combinations will also assist with treatment options and could prolong the time until second-line therapies are required and reduce the risk of resistant strains being transmitted.

While first-line therapy continues to scale-up around Southeast Asia it is important to plan for, and control, the emergence of drug-resistant HIV, particularly as most drug-resistant cases in the future could be ‘hidden’ as minority-resistant variants. Current surveillance programs, which are based around testing newly diagnosed subjects aged less than 25 years rather than genuinely acute infections, will not detect the scale of the problem. Hidden transmitted drug resistance has the potential to drive relatively high levels of drug resistance over the next 5–10 years unless treated cases are monitored regularly and initiate second-line therapies soon after the failure of first-line options. Data from TAHOD suggest that around half of patients beginning ART will require second-line therapies 3 years after beginning treatment [10]. Diagnosing newly acquired infections is important for understanding the true degree of transmitted drug resistance [9], [58] and should be prioritized as we approach the next phase of HIV epidemics in an era of universal treatment access.

While our model is specifically constructed and calibrated to reflect the unique epidemiology of HIV transmission in Southeast Asia, the conclusions drawn from our study can also be applied to other settings. Most countries in Southeast Asia still use d4T-based first-line therapy, which is similar to Sub-Saharan Africa. Access to antiretrovirals is similarly limited in both regions. Our results are generally applicable to non resource-rich settings in which suboptimal regimens are used and there are limited therapeutic options. Our conclusions concerning the dangers of continued use of failed treatment regimens and important value of regular viral load monitoring coupled with access to second-line therapies may assist countries in their scale-up of antiretroviral treatment.

## Supporting Information

Mathematical Details S1 Detailed description of mathematical model.
(0.54 MB DOC)

Figure S1 The seven population subgroups contained within the model. Lines between groups indicate interactions for sexual mixing.
(0.82 MB TIF)

Figure S2 Response surface plot from sensitivity analysis. This plot shows the proportion of cases on ART that have majority-resistant variants (colored contours) versus the rate at which people infected with wild-type acquire drug resistant virus (x-axis) and the average time for majority-resistant variants to emerge for people infected with minority-resistant variants (y-axis) after 20 years of universal treatment access.
(0.32 MB TIF)
