# Supplementary material for: Hidden Drug Resistant HIV to Emerge in the Era of Universal Treatment Access in Southeast Asia
Source: PLoS One. 2010 Jun 8;5(6):e10981. doi: 10.1371/journal.pone.0010981 (PMC2882328; doi:10.1371/journal.pone.0010981)
Supplement: Mathematical Details S1 — Detailed description of mathematical model. (0.54 MB DOC) [file pone.0010981.s001.doc]

**Hidden Drug Resistant HIV to Emerge in the Era of Universal Treatment Access in Southeast Asia**

**Supporting information: Mathematical details**

**Introduction:**

The results presented in the main text are based on a mathematical model. Here we present a full description of the model and a listing of all parameters and assumptions used.

**Model Equations**

HIV disease and transmission dynamics are described with a system of 91 ordinary differential equations, one for each of the 13 structural compartments of our model (Fig. 1) multiplied by seven population subgroups (Fig. S1). These equations are as follows, where subscripts *i*, and *j* are used to denote the population subgroups, Table S1 outlines all parameter values used in the model:

$$\frac{dS_{i}}{dt}=\pi_{i}-S_{i}\left[\mu+c_{i}^{j}\left(\Lambda_{i}^{S}+\Lambda_{i}^{R}\right)\right]$$

$$\frac{dPI_{i}^{S}}{dt}=S_{i}c_{i}^{j}\Lambda_{i}^{S}-PI_{i}^{j}\left(\gamma_{S}^{PI}+\mu+\delta_{S}^{PI}\right)$$

$$\frac{dPI_{i}^{DRD}}{dt}=S_{i}\psi c_{i}^{j}\Lambda_{i}^{R}-PI_{i}^{DRD}\left(\gamma_{R}^{PI}+\mu+\delta_{R}^{PI}+\varphi\right)$$

$$\frac{dPI_{i}^{DRU}}{dt}=S_{i}\left(1-\psi\right)c_{i}^{j}\Lambda_{i}^{R}+\varphi PI_{i}^{DRD}-PI_{i}^{DRU}\left(\gamma_{S}^{PI}+\mu+\delta_{S}^{PI}\right)$$

$$\frac{dCI_{i}^{S}}{dt}=\gamma_{S}^{PI}PI_{i}^{S}-CI_{i}^{S}\left(\gamma_{S}^{CI}+\mu+\delta_{S}^{CI}\right)$$

$$\frac{dTE_{i}^{S}}{dt}=\gamma_{S}^{CI}CI_{i}^{S}-TE_{i}^{S}\left(\tau_{i}+\mu+\delta_{S}^{TE}\right)$$

$$\frac{dT_{i}^{S}}{dt}=\tau_{i}TE_{i}^{S}-T_{i}^{S}\left(\eta+\mu+\delta_{S}^{T}\right)$$

$$\frac{dT_{i}^{DRU}}{dt}=\tau_{i}TE_{i}^{DRU}-T_{i}^{DRU}\left(\mu+\delta_{S}^{T}+\omega\right)$$

$\frac{dT_{i}^{R}}{dt}=\eta T_{i}^{S}+\tau_{i}TE_{i}^{DRD}+\omega T_{i}^{DRU}-T_{i}^{R}\left(\mu+\delta_{R}^{T}\right)$.

Change in the number of uninfected (susceptible) individuals is governed by equation ; the force of infection terms, $\Lambda_{i}^{S}$ and $\Lambda_{i}^{R}$(described below) for seroconverting with wild-type or drug-resistant virus respectively. Once infected, individuals progress into primary HIV infection, governed by equations -. A proportion $\psi$ of individuals infected with a drug-resistant strain will have minority-resistant variants at the time of infection.

Equations - describe the change in the number of individuals in the chronic infection stage of HIV. After the chronic stage, HIV-infected individuals become treatment-eligible (governed by equations -), at which point they may receive ART at rate$\tau_{i}$; we assume that in the era of universal treatment access people will initiate ART within a period of 6 weeks to 1 year of becoming treatment-eligible (see Table S1), although IDUs may take longer to seek treatment because of legal and social barriers. Once on treatment (governed by equations -), we assume that patients will continue using their ART regime, even if treatment failure occurs.

We assume that individuals with majority-resistance strains can become minority-resistant at a rate. This represents wild-type strains becoming the dominant type of virus. We also assume that once on treatment, individuals with minority-resistant strains become majority-resistant under the selective pressure of ART at a rate of$\omega$. Drug susceptible individuals can acquire resistance at a rate of$\eta$.

In all equations, we assume that individuals can leave the sexually active population at a rate$\mu$. Infected individuals can also leave the population due to death caused by HIV. This is represented by$\delta$. We assume different rates for each disease stage, and also differing rates for those infected with drug resistant virus.

**Probability of Transmission**

The transmission probability is based on a number of factors. Disease stage alters the transmission probability according to viral load using the relation [1]:

$\beta_{v}=2.45^{\log(v/w)}\beta_{w}$,

where *w* is the baseline viral load (in our case chronic infection), $\beta_{w}$is the baseline transmission probability, and *v* is the new viral load. Condom use is also a factor affecting transmission. We incorporate condom use in the per partnership calculation based using a binomial calculation [2] in the following equations:

Here, $n$is the number of acts, $\chi$is the proportion of acts using condoms, is the efficacy of condoms, and $\alpha_{2}$is the multiplicative factor affecting drug resistant virus due to reduced fitness.

**Force of Infection:**

The force of infection is governs the rate of new infections based on the transmission probabilities, number of partners, and the number of infected. These are described in equations and:

$$\Lambda_{i}^{S}=c_{i}^{j}\lambda_{S}$$

$\Lambda_{i}^{R}=c_{i}^{j}(\lambda_{DRD}+\lambda_{DRU})$ MERGEFORMAT

Where $c_{i}^{j}$represents the average number of contacts between population group *i* and group *j*, and$\lambda_{S}$,$\lambda_{DRD}$, and $\lambda_{DRU}$are described as follows:

$$\lambda_{S}=\frac{\beta_{v}^{PS}N_{i}^{PS}+\beta_{v}^{CS}N_{i}^{CS}+\beta_{v}^{TES}N_{i}^{TES}+\beta_{v}^{TS}N_{i}^{TS}}{N_{tot}}$$

$$\lambda_{DRD}=\frac{\beta_{v}^{PDRD}N_{i}^{PDRD}+\beta_{v}^{CDRD}N_{i}^{CDRD}+\beta_{v}^{TEDRD}N_{i}^{TEDRD}+\beta_{v}^{TDRD}N_{i}^{TDRD}}{N_{tot}}$$

$$\lambda_{DRU}=\frac{\beta_{v}^{PDRU}N_{i}^{PDRU}+\beta_{v}^{CDRU}N_{i}^{CDRU}+\beta_{v}^{TEDRU}N_{i}^{TEDRU}+\beta_{v}^{TDRU}N_{i}^{TDRU}}{N_{tot}}$$

Here,$\beta_{v}^{S}$, $\beta_{v}^{DRD}$, and $\beta_{v}^{DRU}$refers to the transmission probability associated with drug susceptible, detectible drug resistant, and undetectable drug resistant strains. The additional notations *P, C, TE,* and *T* in front of the viral class groups denote the disease stages, primary, chronic, treatment eligible and treated respectively. The number of infected individuals is denoted by$N_{i}^{S}$,$N_{i}^{DRD}$ and$N_{i}^{DRU}$, using the same convention as for the transmission probabilities. The total population is given by$N^{tot}$.

**Sensitivity Analysis:**

We performed a full uncertainty and sensitivity analysis using SaSAT [3]. We generated 10,000 individual parameter sets via Latin hypercube sampling for the parameters described in Table S1. The model was implemented with Matlab® R2008b. Steady state levels were obtained in the absence of treatment and simulations leading to an overall HIV prevalence between 0.5% and 5% were retained, leaving 2,318 parameter sets. The model was then run to simulate 10 years of the HIV epidemic with full access to ART for treatment-eligible individuals. Partial rank correlation coefficients and factor prioritization by reduction of variance methods were carried out to identify dominant factors, from which response surfaces were generated using multivariate regression analyses.

**Table S1: List of model parameters and their values**

| **Parameter** | **Description** | | | **Value** | | **Ref** |
| --- | --- | --- | --- | --- | --- | --- |
| $1/\gamma_{s}^{PI}$ | Average progression time from primary to chronic infection for individuals infected with drug-sensitive HIV | | | 2-6 months | | [4,5,6] |
| $1/\gamma_{R}^{PI}$ | Average progression time from primary to chronic infection for individuals infected with drug-resistant HIV | | | $\alpha_{1}$. $1/\gamma_{s}^{PI}$ (min 2 months) | | [7] |
| $1/\gamma_{S}^{CI}$ | Average progression time from chronic infection to “treatment-eligible” or AIDS for individuals infected with drug-sensitive HIV | | | 8 – 10 years | | [8,9,10] |
| $\alpha_{1}$ | Multiplier for relative increase in average time for individual infected with drug resistant virus to progress from chronic infection to treatment eligibility, considering reduced viral fitness. | | | 0.75 – 1.5 | | † |
| $1/\gamma_{R}^{CI}$ | Average progression time from chronic infection to “treatment-eligible” or AIDS for individuals infected with drug-resistant HIV | | | $\alpha_{1}\times1/\gamma_{S}^{CI}$ | | |
| IDU_0_ | Percentage of population who are injecting drug users (IDUs) | % Total Pop | | 0.05 –0.5% | [11,12,13] | |
|  |  | % of IDU that are Female | | 10 – 20 % |  |  |
| SW_0_ | Percentage of sexually active women that are sex workers (SWs) | | | 0.5-1% | [14,15] | |
| MC_0_ | Percentage of men who are clients of SWs | | | 5 – 10% | [16,17,18] | |
| MSM_0_ | Percentage of men who are MSM | | | 1-5 % | [18,19,20,21,22,23] | |
| $1/\delta_{S}^{PI}$ | Average rate of AIDS deaths for individuals in Primary Infection class | | | ~0 | ‡ | |
| $1/\delta_{S}^{CI}$ | Average rate of AIDS deaths for individuals Chronic Infection class | | | ~0 | ‡ | |
| $1/\delta_{S}^{TE}$ | Average time to death for untreated individuals with drug-sensitive virus in the AIDS/”treatment-eligible” stage of infection | | | 2 – 4 years | [24,25,26] | |
| $p_{s}$ | Proportion of drug-sensitive treated individuals that achieve viral suppression | | | 0.6 – 0.85 | [27] | |
| $1/\delta_{R}^{TE}$ | Average time to death for untreated individuals with drug-resistant virus in the AIDS/”treatment-eligible” stage of infection | | | $\alpha_{1}\times1/\delta_{S}^{TE}$ years |  | |
| $1/\delta_{S}^{T}$ | Average time to death for treated individuals with drug-sensitive virus in the AIDS/”treatment-eligible” stage of infection | | | $\frac{1}{\delta_{S}^{TE}}<\frac{1}{\delta_{S}^{T}}<15$ | [24,28,29]^a^ | |
| $1/\delta_{R}^{T}$ | Average time to death for treated individuals with drug-resistant virus in the AIDS/”treatment-eligible” stage of infection | | | $\frac{1}{\delta_{S}^{TE}}<\frac{1}{\delta_{R}^{T}}<\frac{1}{\delta_{S}^{T}}$ | [28,30]^b^ | |
| $\eta$ | Percentage of individuals on ART to acquire resistance (have treatment failure) each year | | | 3-5% | [31,32] | |
| $1/\omega$ | Average time for drug resistance to re-emerge upon treatment in individuals that have reservoirs of drug-resistant strains | | | 3 months -$1/\eta$ | [33,34]^c^ | |
| $1/\varphi$ | Average time for virus “reversion” to wild type | | | 0.25 – 5 years | [35,36] | |
| $\psi$ | Proportion of those infected with transmitted drug resistant virus in which resistant viral strain is detectable | | | 0-100% | † | |
| $1/\mu$ | Average time for individuals to be in sexually active population | | | 30 – 35 years | [28,37] | |
| $\beta_{\nu}$ | Probability of transmission via needle sharing per single event, if initial user is HIV-positive | | | 0.005 – 0.01 | [38,39] | |
| $\alpha_{2}$ | Reduction in fitness of drug resistant HIV, decreasing transmission probability of drug-resistant strains | | | 0.05 – 0.5 | [27,40,41] | |
| *w* | Baseline viral load taken at chronic infection | | | 10^4^ – 10^5^ copies/ml | [42,43] | |
| *v*_PI_ | Average viral load at primary infection stage | | | 10^5^ – 10^8^ copies/ml | [5,42] | |
| *v*_CI_ | Average viral load at chronic infection stage | | | 10^4^ – 10^5^ copies/ml | [5,8,42,43] | |
| *v*_TE_ | Average viral load at treatment eligible stage | | | 10^5^ – 10^6^ copies/ml | [10,42] | |
| *v*_T_ | Average viral load at Treated stage | | | 10 – 200 copies/ml | [44,45,46] | |
| $\beta_{w}^{m}$ | Baseline male-to-female transmission probability per act^d^ | | | 0.0001 – 0.002 | [1,47,48,49,50,51,52] | |
| $\beta_{w}^{f}$ | Baseline female-to-male transmission probability per act | | | 0.0001 – 0.0015 | [47,49,50,51,52,53] | |
| $\beta_{w}^{msm}$ | Baseline male-to-male transmission probability per act | | | 0.001 – 0.01 | [49,52,54] | |
| $c_{MC}$ | Number of sexual partnerships per year of males (who are also clients of SWs) | | with SW | 2 – 5 | [55,56] | |
|  |  |  | with GF | 0.5 – 1.5 |  |  |
| $c_{mIDU}$ | Number of sexual partnerships per year of a male injecting drug user | | with SW | 2 – 5 | [55,56] | |
|  |  |  | with GF | 0.5 – 1.5 |  |  |
| $c_{GM}$ | Number of partnerships per year of general males (who are not IDUs nor clients of SWs) | | | 0.75 – 1.5 | [56] | |
| $c_{MSM}$ | Number of partnerships per year of MSM | | | 2 - 6 | [57,58,59,60] | |
| $\chi_{GF}$ | Proportion of acts in which condoms are used for non-SW females | | | 0 – 20% | [56,61,62] | |
| $\chi_{SW}$ | Proportion of acts in which condoms are used by SWs | | | 10 - 50% | [61,63] | |
| $\chi_{MSM}$ | Proportion of acts in which condoms are used by MSM | | | 10 – 50% | [57,64] | |
| $\varepsilon$ | Efficacy of condoms (per act) | | | 80-95% | [54,65,66] | |
| $n_{SW}^{MC}$ | Number of acts per partnership between SW and MC per year | | | 1-6 | [63] | |
| $n_{MC}^{GF}$ | Number of acts per partnership between GF and MC | | | 100 – 150 | [67,68] | |
| $n_{GM}^{GF}$ | Number of acts per partnership between GF and GM | | | 100 – 150 | [67,68] | |
| $n_{needle}^{IDU}$ | Number of times injected drugs per year | | | 300 – 1000 per year | [69,70,71] | |
| $\zeta$ | Percentage of IDUs that share needles | | | 60 – 75% | [72] | |
| $\theta_{needle}$ | Proportion of injections in which sharing IDUs share needles | | | 0 – 1 | † | |
| $\theta_{IDU}$ | Reduction factor in the rate at which IDUs seek treatment, since they are not as likely as other groups. | | | 0 - 1 | † Clinical experience | |
| $1/\tau_{FSW}$ | Average time to receive treatment for SWs | | | 6 weeks – 6 months | Estimate^e^ | |
| $\begin{matrix} 1/\tau_{GF}, \\ 1/\tau_{GM}, \\ 1/\tau_{MC}, \\ 1/\tau_{MSM} \end{matrix}$ | Average time to receive treatment for the non-SWs and non-IDUs | | | 6 weeks – 12 months | Estimate^f^ | |
| $\begin{matrix} 1/\tau_{mIDU}, \\ 1/\tau_{fIDU} \end{matrix}$ | Average time to receive treatment for IDUs | | | $\begin{matrix} 1/\theta_{IDU}\times\\ 1/\tau_{GM} \end{matrix}$ | Estimate^g^ | |
| † Experimental Parameter  ‡ We assume that HIV-infected individuals will not die from AIDS-related illnesses until they have progressed through primary and chronic infection, to the stage of infection of AIDS.  ^a^ The ranges is assumed for those who achieve viral suppression  ^b^ For this range, it is assumed that an individual with a drug resistant virus will survive longer that those without any treatment, but not as long as those with a drug susceptible virus  ^c^ Low range is based on time taken for resistance to re-emerge after structured treatment interruption, and high range based on time for resistance to normally develop  ^d^ Baseline taken at Chronic infection  ^e^ Estimate based on high organisation of this group, with several testing options and locations available  ^f^ Assumed that testing may not occur as regularly for these groups as with FSW  ^g^ We assume that IDUs are less likely to seek treatment immediately due to social and cultural factors | | | | | | |

**References**

1. Gray RH, Wawer MJ, Brookmeyer R, Sewankambo NK, Serwadda D, et al. (2001) Probability of HIV-1 transmission per coital act in monogamous, heterosexual, HIV-1-discordant couples in Rakai, Uganda. The Lancet 357: 1149-1153.

2. Rottingen JA, Garnett GP (2002) The epidemiological and control implications of HIV transmission probabilities within partnerships. Sex Transm Dis 29: 818-827.

3. Hoare A, Regan DG, Wilson DP (2008) Sampling and sensitivity analyses tools (SaSAT) for computational modelling. Theoretical Biology and Medical Modelling 5: 4.

4. Kaufmann GR, Cunningham P, Kelleher AD, Zaunders J, Carr A, et al. (1998) Patterns of viral dynamics during primary human immunodeficiency virus type 1 infection. The Sydney Primary HIV Infection Study Group. J Infect Dis 178: 1812-1815.

5. Richardson BA, Mbori-Ngacha D, Lavreys L, John-Stewart GC, Nduati R, et al. (2003) Comparison of Human Immunodeficiency Virus Type 1 Viral Loads in Kenyan Women, Men, and Infants during Primary and Early Infection. J Virol 77: 7120-7123.

6. Schacker TW, Hughes JP, Shea T, Coombs RW, Corey L (1998) Biological and Virologic Characteristics of Primary HIV Infection. Ann Intern Med 128: 613-620.

7. Pillay D, Bhaskaran K, Jurriaans S, Prins M, Masquelier B, et al. (2006) The impact of transmitted drug resistance on the natural history of HIV infection and response to first-line therapy. AIDS 20: 21-28.

8. Rangsin R, Chiu J, Khamboonruang C, Sirisopana N, Eiumtrakul S, et al. (2004) The natural history of HIV-1 infection in young Thai men after seroconversion. J Acquir Immune Defic Syndr 36: 622-629.

9. Kilmarx PH, Limpakarnjanarat K, Kaewkungwal J, Srismith R, Saisorn S, et al. (2000) Disease progression and survival with human immunodeficiency virus type 1 subtype E infection among female sex workers in Thailand. J Infect Dis 181: 1598-1606.

10. Sabin CA, Devereux H, Phillips AN, Hill A, Janossy G, et al. (2000) Course of viral load throughout HIV-1 infection. J Acquir Immune Defic Syndr 23: 172-177.

11. (2004) Not Enough Graves: The War on Drugs, HIV/AIDS, and Violations of Human Rights in Thailand.

12. Aceijas C, Friedman SR, Cooper HL, Wiessing L, Stimson GV, et al. (2006) Estimates of injecting drug users at the national and local level in developing and transitional countries, and gender and age distribution. Sex Transm Infect 82 Suppl 3: iii10-17.

13. Wattana W, van Griensven F, Rhucharoenpornpanich O, Manopaiboon C, Thienkrua W, et al. (2007) Respondent-driven sampling to assess characteristics and estimate the number of injection drug users in Bangkok, Thailand. Drug Alcohol Depend.

14. Hsieh YH (2002) Changing faces of commercial sex in Thailand: implications for the HIV/AIDS epidemic. J Acquir Immune Defic Syndr 30: 537-540.

15. Matsuda Y (1996) "It's not a Land of Fear and Despair: The HIV/AIDS Pandemic in Thailand". AUICK Newsletter.

16. Kobori E, Visrutaratna S, Kada A, Wongchai S, Ono-Kihara M, et al. (2006) Prevalence and Correlates of Sexual Behaviors Among Karen Villagers in Northern Thailand. AIDS Behav.

17. Jenkins RA, Torugsa K, Mason CJ, Jamroenratana V, Lalang C, et al. (1999) HIV Risk Behavior Patterns Among Young Thai Men. AIDS and Behavior 3: 335 - 346.

18. Liu A, Kilmarx P, Jenkins RA, Manopaiboon C, Mock PA, et al. (2006) Sexual initiation, substance use, and sexual behavior and knowledge among vocational students in northern Thailand. Int Fam Plan Perspect 32: 126-135.

19. van Griensven F, Kilmarx PH, Jeeyapant S, Manopaiboon C, Korattana S, et al. (2004) The prevalence of bisexual and homosexual orientation and related health risks among adolescents in northern Thailand. Arch Sex Behav 33: 137-147.

20. van Griensven F, Thanprasertsuk S, Jommaroeng R, Mansergh G, Naorat S, et al. (2005) Evidence of a previously undocumented epidemic of HIV infection among men who have sex with men in Bangkok, Thailand. AIDS 19: 521-526.

21. Beyrer C, Eiumtrakul S, Celentano DD, Nelson KE, Ruckphaopunt S, et al. (1995) Same-sex behavior, sexually transmitted diseases and HIV risks among young northern Thai men. AIDS 9: 171-176.

22. Kitsiripornchai S, Markowitz LE, Ungchusak K, Jenkins RA, Leucha W, et al. (1998) Sexual behavior of young men in Thailand: regional differences and evidence of behavior change. J Acquir Immune Defic Syndr Hum Retrovirol 18: 282-288.

23. Baxter D (2006) Bangkok’s MSM HIV Explosion – Precursor for Asia’s Mega-cities? HIV Australia 5.

24. Costello C, Nelson KE, Suriyanon V, Sennun S, Tovanabutra S, et al. (2005) HIV-1 subtype E progression among northern Thai couples: traditional and non-traditional predictors of survival. Int J Epidemiol 34: 577-584.

25. Luo K, Law M, Kaldor JM, McDonald AM, Cooper DA (1995) The role of initial AIDS-defining illness in survival following AIDS. AIDS 9: 57-63.

26. Li Y, McDonald AM, Dore GJ, Kaldor JM (2000) Improving survival following AIDS in Australia, 1991-1996. National HIV Surveillance Committee. AIDS 14: 2349-2354.

27. Blower S, Bodine E, Kahn J, McFarland W (2005) The antiretroviral rollout and drug-resistant HIV in Africa: insights from empirical data and theoretical models. AIDS 19: 1-14.

28. Wilson DP, Kahn J, Blower SM (2006) Predicting the epidemiological impact of antiretroviral allocation strategies in KwaZulu-Natal: the effect of the urban-rural divide. Proc Natl Acad Sci U S A 103: 14228-14233.

29. Barbour JD, Hecht FM, Wrin T, Segal MR, Ramstead CA, et al. (2004) Higher CD4+ T cell counts associated with low viral pol replication capacity among treatment-naive adults in early HIV-1 infection. J Infect Dis 190: 251-256.

30. Coetzee D, Boulle A, Hildebrand K, Asselman V, Van Cutsem G, et al. (2004) Promoting adherence to antiretroviral therapy: the experience from a primary care setting in Khayelitsha, South Africa. AIDS 18 Suppl 3: S27-31.

31. Morgan D, Mahe C, Mayanja B, Okongo JM, Lubega R, et al. (2002) HIV-1 infection in rural Africa: is there a difference in median time to AIDS and survival compared with that in industrialized countries? AIDS 16: 597-603.

32. Tang JW, Pillay D (2004) Transmission of HIV-1 drug resistance. J Clin Virol 30: 1-10.

33. Albrecht D, Zollner B, Feucht HH, Lorenzen T, Laufs R, et al. (2002) Reappearance of HIV multidrug-resistance in plasma and circulating lymphocytes after reintroduction of antiretroviral therapy. J Clin Virol 24: 93-98.

34. Ghosn J, Wirden M, Ktorza N, Peytavin G, Ait-Mohand H, et al. (2005) No benefit of a structured treatment interruption based on genotypic resistance in heavily pretreated HIV-infected patients. AIDS 19: 1643-1647.

35. Brenner BG, Routy JP, Petrella M, Moisi D, Oliveira M, et al. (2002) Persistence and fitness of multidrug-resistant human immunodeficiency virus type 1 acquired in primary infection. J Virol 76: 1753-1761.

36. Ghosn J, Pellegrin I, Goujard C, Deveau C, Viard JP, et al. (2006) HIV-1 resistant strains acquired at the time of primary infection massively fuel the cellular reservoir and persist for lengthy periods of time. AIDS 20: 159-170.

37. Vardavas R, Blower S (2007) The Emergence of HIV Transmitted Resistance in Botswana: "When Will the WHO Detection Threshold Be Exceeded?". PLoS ONE 2: e152.

38. Hudgens M, Longini Jr I, Halloran M, Choopanya K, Vanichseni S, et al. (2001) Estimating the Transmission Probability of Human Immunodeficiency Virus in Injecting Drug Users in Thailand. Applied Statistics 50: 1-14.

39. Kaplan EH, Heimer R (1995) HIV incidence among New Haven needle exchange participants: updated estimates from syringe tracking and testing data. J Acquir Immune Defic Syndr Hum Retrovirol 10: 175-176.

40. Blower S, Ma L, Farmer P, Koenig S (2003) Predicting the impact of antiretrovirals in resource-poor settings: preventing HIV infections whilst controlling drug resistance. Curr Drug Targets Infect Disord 3: 345-353.

41. French M, Amin J, Roth N, Carr A, Law M, et al. (2002) Randomized, open-label, comparative trial to evaluate the efficacy and safety of three antiretroviral drug combinations including two nucleoside analogues and nevirapine for previously untreated HIV-1 Infection: the OzCombo 2 study. HIV Clin Trials 3: 177-185.

42. Simon V, Ho DD, Abdool Karim Q (2006) HIV/AIDS epidemiology, pathogenesis, prevention, and treatment. Lancet 368: 489-504.

43. Sarr AD, Eisen G, Gueye-Ndiaye A, Mullins C, Traore I, et al. (2005) Viral dynamics of primary HIV-1 infection in Senegal, West Africa. J Infect Dis 191: 1460-1467.

44. Anekthananon T, Ratanasuwan W, Techasathit W, Sonjai A, Suwanagool S (2004) Safety and efficacy of a simplified fixed-dose combination of stavudine, lamivudine and nevirapine (GPO-VIR) for the treatment of advanced HIV-infected patients: a 24-week study. J Med Assoc Thai 87: 760-767.

45. Bonjoch A, Paredes R, Domingo P, Cervantes M, Pedrol E, et al. (2006) Long-term safety and efficacy of nevirapine-based approaches in HIV type 1-infected patients. AIDS Res Hum Retroviruses 22: 321-329.

46. Yozviak JL, Doerfler RE, Woodward WC (2001) Effectiveness and tolerability of nevirapine, stavudine, and lamivudine in clinical practice. HIV Clin Trials 2: 474-476.

47. Gouws E, White PJ, Stover J, Brown T (2006) Short term estimates of adult HIV incidence by mode of transmission: Kenya and Thailand as examples. Sex Transm Infect 82 Suppl 3: iii51-55.

48. Wawer MJ, Gray RH, Sewankambo NK, Serwadda D, Li X, et al. (2005) Rates of HIV-1 transmission per coital act, by stage of HIV-1 infection, in Rakai, Uganda. J Infect Dis 191: 1403-1409.

49. Royce RA, Sena A, Cates W, Jr., Cohen MS (1997) Sexual transmission of HIV. N Engl J Med 336: 1072-1078.

50. Padian NS, Shiboski SC, Glass SO, Vittinghoff E (1997) Heterosexual transmission of human immunodeficiency virus (HIV) in northern California: results from a ten-year study. Am J Epidemiol 146: 350-357.

51. Leynaert B, Downs AM, de Vincenzi I, for the European Study Group on Heterosexual Transmission of HIV (1998) Heterosexual Transmission of Human Immunodeficiency Virus: Variability of Infectivity throughout the Course of Infection. Am J Epidemiol 148: 88-96.

52. Chesson HW, Pinkerton SD, Voigt R, Counts GW (2003) HIV infections and associated costs attributable to syphilis coinfection among African Americans. Am J Public Health 93: 943-948.

53. Mastro TD, Satten GA, Nopkesorn T, Sangkharomya S, Longini IM, Jr. (1994) Probability of female-to-male transmission of HIV-1 in Thailand. Lancet 343: 204-207.

54. Varghese B, Maher JE, Peterman TA, Branson BM, Steketee RW (2002) Reducing the risk of sexual HIV transmission: quantifying the per-act risk for HIV on the basis of choice of partner, sex act, and condom use. Sex Transm Dis 29: 38-43.

55. Maticka-Tyndale E, Elkins D, Haswell-Elkins M, Rujkarakorn D, Kuyyakanond T, et al. (1997) Contexts and patterns of men's commercial sexual partnerships in northeastern Thailand: implications for AIDS prevention. Soc Sci Med 44: 199-213.

56. Rongkavilit C, Naar-King S, Chuenyam T, Wang B, Wright K, et al. (2007) Health risk behaviors among HIV-infected youth in Bangkok, Thailand. J Adolesc Health 40: 358 e351-358.

57. (2006) HIV prevalence among populations of men who have sex with men--Thailand, 2003 and 2005.: Centers for Disease Control and Prevention (CDC).

58. Jiang J, Cao N, Zhang J, Xia Q, Gong X, et al. (2006) High prevalence of sexually transmitted diseases among men who have sex with men in Jiangsu Province, China. Sex Transm Dis 33: 118-123.

59. Colby DJ (2003) HIV knowledge and risk factors among men who have sex with men in Ho Chi Minh City, Vietnam. J Acquir Immune Defic Syndr 32: 80-85.

60. Liu H, Yang H, Li X, Wang N, Liu H, et al. (2006) Men who have sex with men and human immunodeficiency virus/sexually transmitted disease control in China. Sex Transm Dis 33: 68-76.

61. Punpanich W, Ungchusak K, Detels R (2004) Thailand's response to the HIV epidemic: yesterday, today, and tomorrow. AIDS Educ Prev 16: 119-136.

62. UNDP (2004) Thailand’s response to HIV/AIDS: progress and challenges. Bangkok.

63. Buckingham RW, Moraros J, Bird Y, Meister E, Webb NC (2005) Factors associated with condom use among brothel-based female sex workers in Thailand. AIDS Care 17: 640-647.

64. Mansergh G, Naorat S, Jommaroeng R, Jenkins RA, Stall R, et al. (2006) Inconsistent condom use with steady and casual partners and associated factors among sexually-active men who have sex with men in Bangkok, Thailand. AIDS Behav 10: 743-751.

65. Cayley WE, Jr. (2004) Effectiveness of condoms in reducing heterosexual transmission of HIV. Am Fam Physician 70: 1268-1269.

66. Davis KR, Weller SC (1999) The effectiveness of condoms in reducing heterosexual transmission of HIV. Fam Plann Perspect 31: 272-279.

67. Whitehead SJ, Kilmarx PH, Blanchard K, Manopaiboon C, Chaikummao S, et al. (2006) Acceptability of Carraguard vaginal gel use among Thai couples. AIDS 20: 2141-2148.

68. Tovanabutra S, Robison V, Wongtrakul J, Sennum S, Suriyanon V, et al. (2002) Male viral load and heterosexual transmission of HIV-1 subtype E in northern Thailand. J Acquir Immune Defic Syndr 29: 275-283.

69. Perngmark P, Celentano DD, Kawichai S (2003) Risk factors for HIV infection among drug injectors in southern Thailand. Drug Alcohol Depend 71: 229-238.

70. Choopanya K, Des Jarlais DC, Vanichseni S, Kitayaporn D, Mock PA, et al. (2002) Incarceration and risk for HIV infection among injection drug users in Bangkok. J Acquir Immune Defic Syndr 29: 86-94.

71. Vanichseni S, Kitayaporn D, Mastro TD, Mock PA, Raktham S, et al. (2001) Continued high HIV-1 incidence in a vaccine trial preparatory cohort of injection drug users in Bangkok, Thailand. AIDS 15: 397-405.

72. Perngmark P, Celentano DD, Kawichai S (2003) Needle sharing among southern Thai drug injectors. Addiction 98: 1153-1161.
