# Supplementary figures and images for: Hidden Drug Resistant HIV to Emerge in the Era of Universal Treatment Access in Southeast Asia
Source: PLoS One. 2010 Jun 8;5(6):e10981. doi: 10.1371/journal.pone.0010981 (PMC2882328; doi:10.1371/journal.pone.0010981)

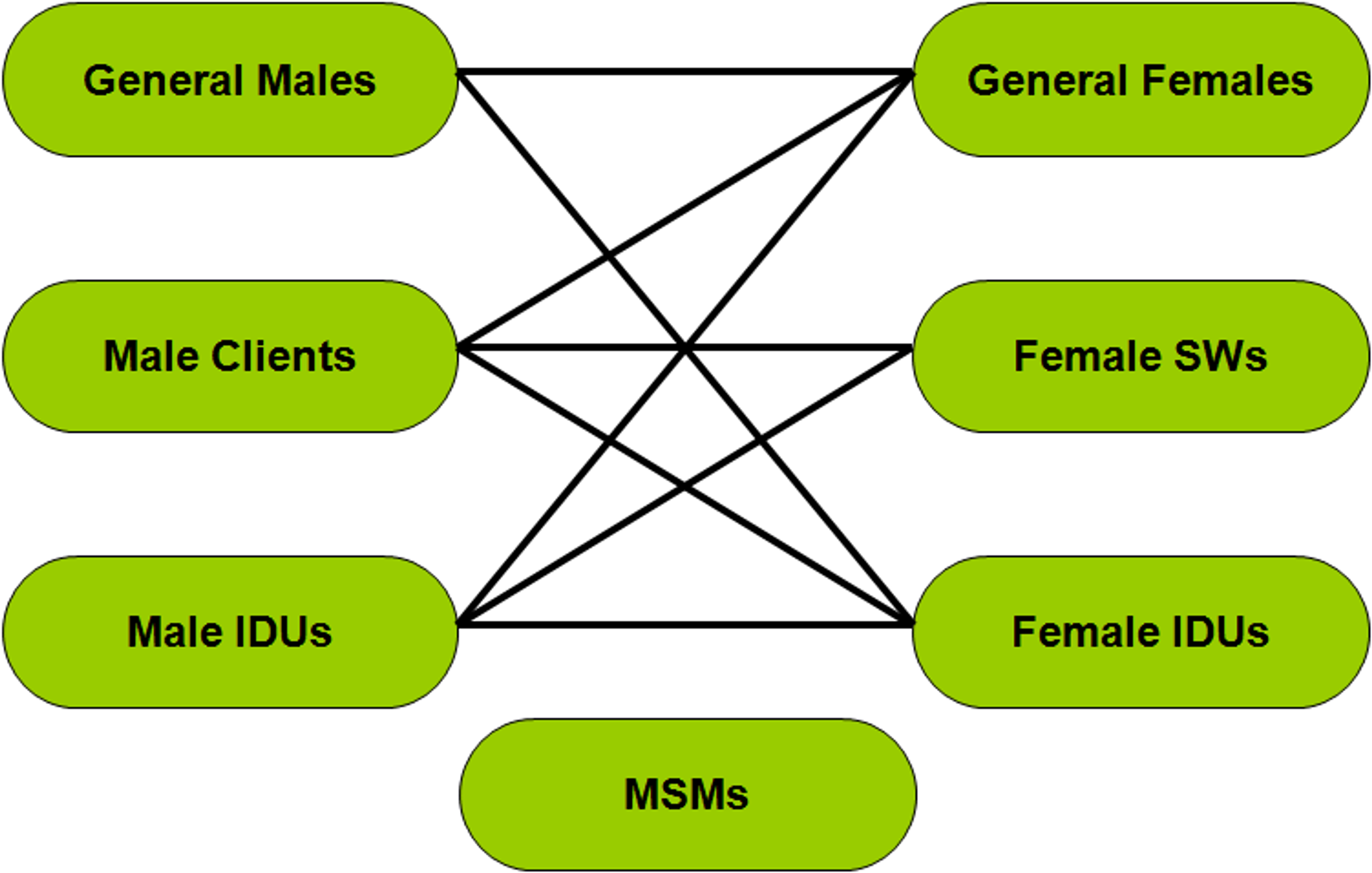

Supplement: Figure S1 — The seven population subgroups contained within the model. Lines between groups indicate interactions for sexual mixing. (0.82 MB TIF) [file pone.0010981.s002.tif]

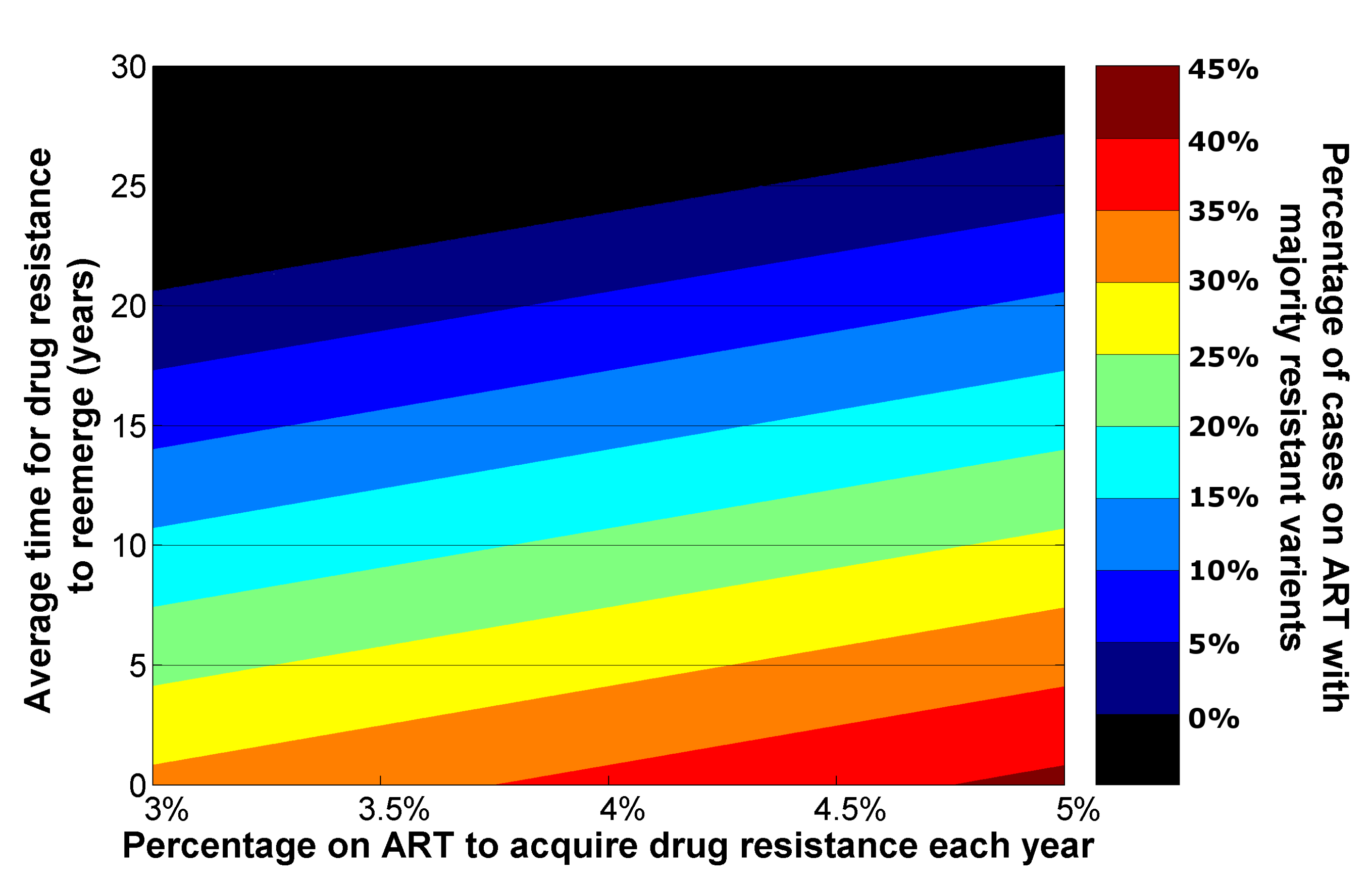

Supplement: Figure S2 — Response surface plot from sensitivity analysis. This plot shows the proportion of cases on ART that have majority-resistant variants (colored contours) versus the rate at which people infected with wild-type acquire drug resistant virus (x-axis) and the average time for majority-resistant variants to emerge for people infected with minority-resistant variants (y-axis) after 20 years of universal treatment access. (0.32 MB TIF) [file pone.0010981.s003.tif]
